# Cholera Nostras and Cholera Asiatica

**Published:** 1897-07-24

**Authors:** T. Lauder Brunton

**Affiliations:** a Clinical Lecture delivered at St. Bartholomew's Hospital, on Friday


### Medical Progress and Hospital Clinics.

[*The Editor will be glad to receive offers of co-operation and contributions from members of the profession. All letters should be addressed to* The Editor, at the Office, 28 & 29, Southampton Street, Strand, London, W.C.]

### CHOLERA NOSTRAS AND CHOLERA ASIATICA.

By T. Lauder Brunton, M.D., F.R.S., a Clinical Lecture delivered at St. Bartholomew's Hospital, on Friday, July 9th, 1897.

To-day I propose to draw your attention to cases of what is termed cholera nostras. There are two kinds of cholera which are usually distinguished from one another, namely, the Asiatic form and the European form or cholera nostras. The main difference between these two kinds is said to be the occurrence in Asiatic cholera of pure rice-water motions, untinged in the very least by bile. The motions look almost like rice-water; they are quite colourless, with only a slight whitish opacity and sometimes flakes of mucus present in them. In cholera nostras, on the other hand, the motions may be quite fluid, but they are generally more or less tinged with bile or with fæculent matter. As I shall have to mention, however, this clinical distinction does not always hold, and I know that it does not hold because I had an experience of cholera nostras in my own person some four months ago. I went to bed perfectly well. Between one and two o'clock I awoke and felt sick. I then was seized with violent vomiting, so violent that the whole of the contents of the stomach were brought up, and then followed mucus tinged with blood. Now you do not generally get mucus becoming tinged with blood unless the vomiting is very violent. While the vomiting was still continuing I was seized with a desire to go to the closet, and then the motions were somewhat of the same character as the vomiting, namely, exceedingly violent, coming away in violent gushes. The evacuations were perfectly fluid, and at first consisted of fluid fæcal matter, but the fæcal colour disappeared, and finally the motions were absolutely colourless. They were just like rice-water, and, in fact, were precisely similar to the motions that we find in Asiatic cholera; there were, however, no cramps.

There was comparatively little pain in the abdomen, and as soon as the vomiting and purging had ceased, I fell asleep and awoke after two or three hours' rest with no further symptom excepting excessive weakness. This, however, was not so great as to prevent me from going through my ordinary day's work. In my case, therefore, the accession of the symptoms was very sudden; they were very severe while they lasted, but they were local and not general. There was vomiting and purging with great violence, but there was no cramp, no abdominal pain, and there was not the excessive prostration that you usually find in cases either of cholera nostras or Asiatic cholera. I took no remedy, excepting that on the next morning I had a little bicarbonate of soda and bismuth, so as just to quiet a little nausea that still remained, and I had no further return of the symptoms. My case, therefore, shows that the one clinical symptom that may be looked upon as distinctive does not differentiate completely between Asiatic cholera and cholera nostras.

The great majority of cases of cholera nostras recover, but they do not always do so—at least, they do not if we accept as a distinction between cholera Asiatica and cholera nostras the fact that the motions contain bile. Some years ago there was an epidemic of cholera in London, not a very great one, but still there were a good many cases. At that time I was called to see a patient living at Kew. He was a gentleman with no definite occupation, and amused himself a good deal by boating upon the river. He had taken his wife up in a boat towards Oxford, and after they had gone part of the way he got out and was towing the boat. In doing this he had to cross a field which was watered and manured. The smell was exceedingly powerful and very disagreeable, and, as he expressed it, "It seemed to get about his heart." It made him faint and sick. He continued to tow for a while, then got into the boat again and went on a little further till he came to a public-house. He sent for some beer, thinking that this would quiet his stomach, but he became violently sick immediately, and continued suffering more or less for a day or two. He came back to Kew, and I then saw him. He was suffering from violent sickness and violent diarrhœa. His face was getting thin and shrunken, his colour was slightly bluish, the surface of his skin was cold, his pulse was very feeble, and he had marked cramp in the extremities, but the motions, although perfectly liquid, were always tinged slightly with bile. In spite of all I could do, however, the man's strength gradually diminished, and he sunk and died; and although one might be inclined to say, from the occurrence of other cases of Asiatic cholera in London about the same time, that this was a case of Asiatic cholera, yet the clinical difference, the colourless stools, which is usually recognised as being the typical symptom of Asiatic cholera, was absent in his case, whereas it was present in my own case, and yet I had no further severe attack. You see, therefore, that we cannot rely upon this clinical difference between the two kinds of cholera, and I do not know that there is any other difference upon which you can rely, because, as you see, cholera nostras may present all the symptoms of Asiatic cholera excepting the one, and it may yet prove fatal, and you may find the characteristic symptoms of cholera Asiatica in a case of cholera nostras, and yet the patient may recover perfectly well.

Now the sudden occurrence of the symptoms in cholera nostras is usually rather characteristic. We may, however, have the symptoms occurring less suddenly than in the cases just described, or in the one which I am about to read to you. This case was that of Michael E., a labourer, aged 19, who was admitted on July 1st, 1897. He was admitted in a state of collapse. He had been at his usual work and in good health on the morning of July 1st, that is on the morning of the day on which he was admitted. Whilst working he felt sick, and vomited, and at the same time was seized with profuse diarrhœa. Both the vomiting and the purging lasted for two hours, during which time no one was aware of his condition. He was then assisted upstairs and brought to the surgery. When he arrived there he was in a condition of collapse with cold skin, blue lips and ears; his temperature was 95 deg.; his face was drawn and shrunken, the pulse was weak and was only partially felt at the wrist. He complained a good deal of abdominal pain, and the fingers and knees were cramped and stiff. He drank water, but shortly afterwards vomited it again in a somewhat peculiar way; it was ejected with such great violence as to pass nearly eight feet across the room in which he was then lying. His respirations were sighing. He had always been a healthy man, and was said to have had no fever or other illness. There was no history of his having partaken of unsound food, so that the causation of the attack could not be traced. The family history was good. After admission he began to improve, but when I saw him in the middle of the day his condition, although rather better than it was when he was admitted, was still very bad indeed. His face was still drawn, skin still cold, the abdomen was retracted, and the respiration was still sighing. He had passed a motion of a peculiar character; it looked almost like frog's spawn, but of a curious colour. It was a sort of pinkish-brown colour, and probably consisted of mucus. On account of the irritability of his stomach and intestines, I did not think that there was much chance of anything given either by the mouth or rectum being absorbed, and I consequently ordered him a subcutaneous injection of one-hundredth of a grain of atropine. This was ordered so as to neutralise the poison which I supposed to have been acting upon him, and to be producing the symptoms. At the same time he was ordered to have an enema of water simply coloured with permanganate of potash, in order to destroy any bacilli that might be present in the intestine, and that might be likely to manufacture more poison, as well as possibly to destroy any alkaloids that might be present in the intestine itself. After this injection he had no further diarrhœa, and after a second injection of atropine he was very much improved indeed, and about eight p.m. on the same night he was almost well. The face became full, the colour good, and the pulse also became full and regular.

The next case is that of a man, Arthur M., aged 17, who was admitted on June 15th. Four days before admission he had been suffering from headache, pain in the epigastrium, and diarrhœa. These symptoms continued till admission, and then he had vomiting also. When I saw him he was complaining of severe headache and pain all over the abdomen; his face was drawn, his eyes were sunken, his temperature was 96°, and yet he had a pulse of 120 per minute. The tongue was covered with a very thick coating, the bowels had been very loose, the spleen was not felt, and the abdomen was not tender. He was treated in somewhat the same way as the previous case, but not precisely. The first thing that was done with him was to try and eliminate from the intestines both the poison and the microbes which might be forming it; and in order to do this two drachms of castor oil were administered, and along with this were given ten minims of tincture of opium in order to quiet the intestine, and after that he had twenty-five minims of tincture of belladonna with the compound bismuth draught. He had also belladonna fomentation to the abdomen. The next day the dose of belladonna was increased to ten minims every four hours, and this was continued up to June 21st. By this time the patient's symptoms had nearly all passed away, and he was feeling perfectly well; on the 25th he got up, and on the 27th he was discharged cured.

Now these symptoms are very like those produced by certain classes of poisons, both mineral and vegetable. The best example of such a mineral poison is arsenic, and of a vegetable poison poisonous mushrooms. Those two kinds of poison give rise to symptoms very much like those that we have found in the cases I have just described, but in them we could not trace either arsenic or poisonous mushrooms. In fact, we found it impossible to trace any cause for the symptoms. At the same time, we may conclude that these symptoms were due to some kind of poison having an action very much like that of poisonous mushrooms.

Many years ago I pointed out that the symptoms of Cholera Asiatica were almost identical with those produced by muscarine, the poisonous alkaloid obtained from poisonous mushrooms, and I then suggested that the administration of atropine might prove useful as a means of antagonising the cholera poison, just as it would antagonise the poison of mushrooms. It was on account of the similarity between the symptoms occurring in the cases which I have mentioned, that I tried the belladonna and the atropine; belladonna in the one case where I supposed it might be absorbed because the symptoms were not so severe, and the atropine in the other case where absorption from the stomach or intestines seemed to be very unlikely on account of the violence of the vomiting and the purging.

Now it was found some years ago that during the decomposition of albuminous matters various poisons are formed, namely, choline, neurine, and muscarine. It is quite probable then that the cause of the symptoms observed in our cases was actual poisoning. These people were poisoned by an alkaloid just as distinctly as if they had swallowed a lot of poisonous mushrooms and been killed or made ill by the poison they contained; but in them the poison was probably not preformed in the food which they took, but was actually formed in the intestines of the patient by decomposition of albuminous matters produced by bacteria. We know but little about the bacteriology of cholera nostras, and even the pathology of Asiatic cholera has not been fully determined. Every one is now agreed, I think, that the symptoms of Asiatic cholera are due to some toxin, and this toxin is formed by the agency of certain bacilli and more especially by the so-called comma bacilli of Koch, but this comma bacillus has not a monopoly of producing Asiatic cholera. It generally seems to be present; but D. D. Cunningham found in a number of cases, observed and collected, that, although the clinical symptoms were identical, the cholera bacillus was not to be found in the dejecta of the patients, and he found various other bacilli. He separated no less than eleven kinds, all of which might fairly claim to be cholera bacilli. Now, in the case of cholera nostras, we know, perhaps, even less about the bacteriology of the disease than we do in the case of cholera Asiatica. There are two bacilli that are said to have caused cholera nostras; the one is known as Finkler-Prior, which has an appearance something like the ordinary comma bacillus, and you will see from the specimen under the microscope that it has a slightly curved appearance. The other one was found by Dr. Klein to be present in milk, and this bacillus is not destroyed even by boiling the milk, because it forms spores which may resist the boiling unless this be fairly complete, and unless it be repeated so that the milk is actually sterilised. Dr. Klein has given to it the name of Bacillus enteriditis sporogenes, on account of its causing enteritis, and having the power of producing spores. Whether either of those is the real cause of the decomposition of albuminous matters, and the formation of toxins and muscarine, remains a subject for further investigation; but, at any rate, that is all we know about it up to the present moment.

The prognosis in cases of this sort is generally good, but, as I told you when speaking of the case at Kew, they do not always get well. They may die, but in the great majority they recover; and even in the very severe case of Michael E., the patient was, apparently, beginning to recover even before he got the atropine, although I think that the injection of the atropine probably helped him very considerably, and perhaps made all the difference in his case, because when I saw him before the injection he was still very ill, so ill that I thought it very doubtful indeed whether he would get round.

Then the treatment of such cases. First of all, the prophylactic treatment. You can readily see that if you get pure albuminous materials without the bacilli into the intestine they will be digested in the ordinary way, and will probably give rise to no mischief; that if you give non-nitrogenous materials such as bread and butter along with the bacilli, the bacilli will not have the material to work upon, and they will not be able to form those toxins, and so again you may get no result. But if you get albuminous materials together with the bacilli, then very likely these alkaloidal bodies will be formed, and poisoning will occur. Very probably it is because one is so very apt to get a number of bacilli introduced into the body that diarrhœa is so general during summer, because there are a great many materials which are often consumed during summer which contain bacteria, and probably they are very numerous and of different kinds. For example, to give an illustration of what I mean, if any of you were to take a meal containing, let me say, steak and bread and butter, or a potato, then you would be all right because this would be all thoroughly cooked, and there would be no decomposition of the albuminous materials. Supposing, again, you take a meal of tea, bread and butter, and some fruit which is over-ripe and decaying, containing, let us say, a number of microbes; again no harm will be done because the tea, the bread, and the butter do not afford the microbes the material from which they may split off these toxins. If, however, you take a mixed meal containing meat or eggs along with the over-ripe and decaying fruit, the meat or the eggs will afford an albuminous material, from which the bacteria contained in the decayed fruit may split off toxins, and then you may get a case of cholera nostras. In order to avoid it then, one great thing is to avoid over-ripe fruit, unless this has been thoroughly cooked; and one sees that people generally are aware of the danger because they are chary of taking over-ripe fruit, knowing that consuming such is apt to lead to pain and diarrhœa. Then this peculiar bacterium that Dr. Klein investigated apparently occurs very much in milk, and even when the milk has been curdled it is contained in the whey, and if the whey be boiled it will still prove poisonous, because the spores are still present in the whey, and when swallowed they develop and yield the bacteria which caused the poisoning. One therefore requires to be exceedingly careful about milk in hot weather, and sometimes it may be possible to trace the occurrence of cases of cholera nostras to milk or cream. I did not know about the researches of Dr. Klein upon this bacterium when I was investigating the case of Michael E., otherwise I might have made more careful inquiries as to whether he had not had some milk in the morning or evening before admission. We did not go carefully into that question, but had we done so we might have found the cause of the disease in this case.

In regard to treatment, this divides itself into two parts. The first is to evacuate any bacilli that may be in the stomach or intestines, and to kill them if you possibly can by the use of disinfectants. The second part is to neutralise the poisons which are giving rise to the symptoms. Now in my own case the bacteria seemed to have become evacuated simply owing to the profuse secretion by the bowel. By the violent movements in vomiting probably most of the mucus that lines the stomach was evacuated and most of the bacilli with it, and owing to the profuse secretion from the intestine the bowel was completely washed out and the bacteria seemed to have passed out along with it, so that there was no further disturbance. But in cases where this does not occur you often wash the bowel out thoroughly by giving some substance that is likely to clear it out without giving rise to irritation, and more especially castor oil is preferred in these cases because it does not tend to cause any special irritation. One would imagine that salts ought to be very efficient in the treatment of these cases, but practically Epsom salts and other saline purgatives do not seem quite so satisfactory as castor oil. The castor oil seems to clear the bowel out thoroughly, and the salts seem to tend to increase the intestinal secretion, which the poison itself has already stimulated to quite a sufficient extent. One of the best modes of treatment, therefore, is to give a dose of castor oil and along with it five to ten minims of tincture of opium, so that, after the bowels have been cleared out by the castor oil, you may have the residual sedative tendency of the opium remaining behind, which tends to check the painful griping and to check the peristaltic movements of the intestine, so as to stop both pain and purging. In the case of Michael E., where the danger seemed to me great, I thought that probably the best plan was to wash the bowel out with a large enema, tinging it simply with a little permanganate of potash in order to destroy bacteria and any toxins; and this seemed to answer very well, because the patient had no diarrhœa after the enema was evacuated. Then in regard to the counteracting of the effect of any poison that has been formed, I believe that belladonna and atropine are the two best things; that they are better even than opium. The advantage of opium is that it lessens the pain so greatly, and it also tends to check purgation. But purgation in such a case as this probably depends upon a definite poison, and if we can find a drug that will antagonise the poison we shall gain our end much more readily than if we simply use another drug which has a sort of general sedative action but does not completely antagonise the poison. Opium or morphine has the general effect of soothing the intestine, but neither of them antagonises muscarine in the same way that atropine does. Therefore, I think that in cases of cholera Asiatica, or cholera nostras, atropine and belladonna probably form the best means that one can adopt for the purpose of counteracting any poison.

